# Hot take on a cool topic: is there really a need for a systemic master mediator of metabolic suppression in mouse daily torpor?

**DOI:** 10.12688/wellcomeopenres.27516.1

**Published:** 2026-08-28

**Authors:** Mike Ambler, Anthony Pickering, Steven Swoap

**Affiliations:** 1 School of Psychology & Neuroscience, University of Bristol, Bristol, Select..., BS8 1TD, UK; 2 Department of Biology, Williams College, Williamstown, Massachusetts, USA

**Keywords:** torpor, hibernation, metabolism, body temperature

## Abstract

We argue in this perspective that daily torpor in the mouse, induced by a lack of caloric intake and a relatively cool environment, does not require a dedicated “master mediator” for systemic metabolic rate (MR) suppression. Instead, a set of centrally mediated changes, most notably: cessation of physical activity; dramatically reduced thermogenesis, along with a lowering of the defended minimum body temperature (Tb); and, reduced cardiovascular work, together drive the substantial drop in metabolic rate at the initiation of daily torpor in the mouse. That is, rather than invoking a global, organism-wide suppression of metabolism across all tissues, we argue that MR reduction during mouse daily torpor is selective and physiologically organized, targeting a small number of energetically dominant organ systems. Once Tb has declined in response to the initial physiological adaptations, temperature-dependent effects on biochemical reaction rates can account for much of the further reduction in whole-animal MR during the torpid state, resulting in an ultimate fall in MR of 75–80% in daily torpor of the mouse. This framework also aligns torpor with the normal ultradian rhythm of sleep-associated reductions in MR and Tb, differing mainly in the much deeper nadir reached.

## Plain Language summary

The question: When a mouse goes into daily torpor (a short-term, deep energy-saving state triggered by fasting or cold), does the body need some kind of master “off switch” that shuts down metabolism everywhere at once — the way many researchers have assumed for hibernators?

The authors’ answer: no, not for mice. They argue you don’t need to invoke any special whole-body metabolic suppressor. Instead, the big drop in metabolic rate during mouse daily torpor can be fully explained by a handful of ordinary, centrally controlled changes plus simple physics:

1. Getting less active — the mouse stops moving and stops digesting food. This saves some energy, but most of that saving already happened when it fell asleep, before torpor even started.
2. Turning down heat production — the mouse’s brown fat (its main heat-generating tissue) is switched down via the nervous system. This is a bigger saving — roughly a quarter to over half of its active-state energy use.
3. Slowing the heart down — heart rate falls substantially (partly nerve-driven, partly just because the heart itself gets cold), saving at least another 10%.
4. The temperature effect on chemistry itself (Q10) — once the mouse stops defending a normal body temperature, its temperature drops (in mice, often to 25 °C or lower). Cooler tissue means every enzyme in every cell runs more slowly — no active signal required, just basic biochemistry. The authors calculate this alone could account for roughly 60% of the total energy savings.

Add these up and you get the observed 75–80% drop in energy use during torpor — without needing to propose a special systemic mechanism that actively “turns off” cells everywhere.

When we consider the scale of endotherms, to state the obvious, the mouse is small. Similar creatures to the mouse, like the shrew and hummingbird live on the edge of existence. Their small size results in a massive surface area-to-volume (SA/vol) ratio. For example, with a surface area of 75 cm ^2^ ( Dawson, 1967) and a body weight of 25 g, the SA/vol ratio of the mouse is some 14x higher than that of a human. Because heat loss occurs across the surface area while heat is generated by body volume (a surrogate for tissue mass), up to 30% of total energy expenditure is directed to thermogenesis in the mouse at 22 °C ambient temperature ( Reitman, 2018). Hence, small endotherms face a precarious balance when food is scarce. Their lives are centered on acquiring and storing energy, through foraging, eating, caching and fat-accumulation, to fuel the heat-generating engines. Since such animals only have limited space for fat reserves, even a short fast can be disastrous. As a result, in the absence of food, many small mammals suspend ‘normal’ homeostasis to reduce their metabolic demands. This reduced metabolism results in the state of daily torpor, where most physiological activities are reduced. Indeed, if a fasted mouse were to maintain the high MR required for normal homeothermy, it would rapidly exhaust its limited energy reserves. In this context, torpor is a protective strategy that prevents catastrophic energy depletion.

In contrast, when exposed to cool ambient temperatures in the presence of food, mice increase intake and elevate their metabolic rate, to maintain body temperature ( Hudson & Scott, 1979). Hence, bouts of daily torpor can be prevented by hormonal signaling that indicates the presence of fuel, either as stored fat, a recent meal, or the expectation of a meal, allowing the heat producing engines the fuel needed to defend a ‘normal’ minimum T _b_ ( Gavrilova et al., 1999; Van Der Vinne et al., 2018). Further, fasted mice given the choice between a cool or warm environment preferentially choose the warm one, thereby avoiding torpor ( Craig et al., 2021). As such daily torpor in the mouse is a physiological response of last resort.

The metabolic benefits of torpor can be enormous, even lifesaving ( Geiser & Brigham, 2012; Koizumi et al., 1996; Lyman et al., 1981). The metabolic rate during torpor is well below even the basal metabolic rate (the MR from cellular activities in the absence of physical activity and thermoregulatory heat production), resulting in substantial energy savings up to 75–80% in the mouse ( Geiser, 2004; Hudson & Scott, 1979; Nestler, 1990b; Ruf & Geiser, 2015). This raises the important question of how these small mammals achieve the energy savings of daily torpor: is there a global signal that triggers widespread suppression of cellular metabolism in response to food deprivation in the mouse?

Torpor has long been assumed to require an “active system-wide metabolic suppression” ( Geiser, 1988a, 2004; Heldmaier et al., 2004). Indeed, there is a great deal of evidence from many mammals entering torpor that MR falls before T _b_, including mice ( Nestler, 1990a, 1990b; Udoh et al., 2024), suggesting an active metabolic suppression. This is not a point of dispute. Rather, we argue here that the daily torpor seen in a mouse does not require a solution that invokes *systemic* suppression of metabolism. We argue that metabolic suppression during mouse daily torpor is selective and physiologically discretely coordinated, targeting only a small number of energetically dominant organs. Indeed, only a few targeted core physiological changes, initiated centrally (i.e. by the brain) that modulate organ-level metabolism are sufficient to suppress metabolism to the level needed to initiate torpor in the mouse. Those key allostatic changes to move from an active state, initially to a sleep state and thence to a torpid state are 1) cessation of physical activity, 2) decreased thermogenesis in a cool environment via altered autonomic outflow, 3) decreased cardiorespiratory work mediated via the autonomic nervous system and the respiratory control network, and 4) cessation of metabolic activity associated with food intake and digestion. Once those changes have been made and the animal no longer defends a euthermic T _b_, significant and rapid reductions in oxygen consumption would be achieved. Subsequently, the continued decline in MR and T _b_ follow passively from the temperature-dependence of underlying biochemical reactions. Collectively these alterations of regulatory set points and behavioural changes are likely to be sufficient to achieve the target of energy conservation without the need to invoke a separate process that switches off metabolism at the cellular level (such as via mitochondrial inhibition).

Ultradian rhythms are those behavioural/physiological changes that occur more frequently than once per day and are seen in species throughout the animal kingdom ( Blessing & Ootsuka, 2016; Meyer et al., 2012; Škop et al., 2024). Mice display such ultradian rhythms in metabolic rate, T _b_, heart rate, activity, and blood pressure every 2–3 hours corresponding directly with the wake/sleep cycle ( Škop et al., 2024). When the mouse is awake, it 1) is physically active, 2) has brown adipose tissue (BAT) activation via the sympathetic nervous system, and 3) has an autonomically-mediated increased heart rate and blood pressure, resulting in an elevated metabolic rate and a higher defended minimum T _b_. Metabolic rate and T _b_ drop substantially at the end of these active periods. When the mouse sleeps, it achieves a lower metabolic rate through cessation of physical activity; reduced cardiac work; lower BAT activity; and a lower defended T _b_. Because of its small size and the resultant thermal lability ( Gordon, 2012), simply decreasing physical activity, slowing heart rate, and decreasing BAT activity results in a 1.5–2 °C T _b_ drop between the active state and sleep state ( Škop et al., 2024). It appears that the mouse defends this new lower T _b_ - presumably through continued partial activation of heat generation pathways.

Torpor bouts reliably occur at the end of an ultradian “active state” (and often a “hyper-active” state), and typically near the end of the dark phase ( Van Der Vinne et al., 2018). Mice enter torpor through a sleep state ( Huang et al., 2021; Lo Martire et al., 2020), and so some metabolic savings occur when the mouse falls asleep but before torpor starts. We hypothesise that further changes in those same parameters (physical activity, degree of cold-induced thermogenesis, cardiac work) plus the additional impact of lower T _b_ on enzymatic activity is all that is needed to explain the fall in metabolic rate seen in mouse daily torpor. Let’s parse the role of each one of those parameters in the contribution to a torpid metabolic rate, starting with physical activity.

## Reduced physical activity: moderate metabolic gains when moving from sleep to torpor

When mice fall asleep, they obviously become inactive, so there are limited metabolic savings from reduced physical activity in the torpid mouse beyond the gain from falling asleep. However, the additional reduction in respiratory rate from ~100–150 breaths/minute when asleep to ~10–20 breaths/minute in torpor would allow some further metabolic savings ( Morhardt, 1970). Similarly, while a sleeping fed mouse continues to expend energy on digestion, a fasted torpid mouse has minimal food in its gastrointestinal tract, so there would be some metabolic savings from this in torpor.

## Reduced thermogenic activity: greater than 25% saving of active metabolic rate

Non-shivering thermogenesis, unlike physical activity, is greatly reduced when moving from sleep to torpor. The sleeping mouse has some thermogenic activity (dependent upon the ambient temperature), likely from heat generation from brown fat and/or skeletal muscle that defends their T _b_ while asleep ( Szentirmai & Kapás, 2014). Therefore, there is room to reduce metabolic expenditure should the mouse no longer defend a T _b_ of ~35 °C. Non-shivering thermogenesis alone can contribute up to 60% of the total metabolic rate in the cold-adapted and euthermic mouse ( Cannon & Nedergaard, 2011). The mouse saves about 25% of total energy expenditure when decreasing BAT activity as it moves from the active state to sleep state ( Škop et al., 2024). Based on these data, the savings the mouse gained by moving from sleep to torpor would be between 25 to 60% of an active mouse’s metabolic rate.

## Reduced cardiac work: at least 10% saving of active metabolic rate

Torpor reduces cardiac work by reducing heart rate and cardiac output. During sleep the mouse heart rate reduces from ~650 to ~475 beats per minute and mean arterial pressure from ~125 to ~105 mmHg, which has been estimated to result in a 6% reduction in metabolic rate ( Škop et al., 2024). During entrance into daily torpor in the mouse, and before any significant change in T _b_, heart rate and mean arterial pressure reduce further compared to sleep, with a fall to ~275 bpm and ~ 80 mmHg, respectively ( Swoap & Gutilla, 2009). Indeed, it has been long known that the parasympathetic drive to the heart is strongly engaged at entrance into torpor ( Harris & Milsom, 1995; Morhardt, 1970; Williams et al., 2002). Further, the direct cooling effect on the cardiac pacemaker function will serve to only increase energetic savings, as heart rate and mean arterial pressure continue to fall to a minimum of 70 bpm and 60 mmHg at the temperature nadir of the torpor bout in the mouse ( Swoap & Gutilla, 2009). The slowing of heart rate and lowering of blood pressure reduces cardiac work, which we estimate to be an additional 10% of the active metabolic rate at the onset of torpor.

## Reduced enzyme activity (Q _10_ effect): 8% of active metabolic rate per °C

We propose here that the effect of a reduced T _b_ on the rate of enzymatic reactions within cells makes up the lion’s share of the savings. We are by no means the first to make this suggestion ( Geiser, 1988a; Heldmaier & Ruf, 1992; Staples et al., 2022). This was a hotly debated topic in the hibernation community a few decades ago. Active systemic suppression of metabolism beyond physiological adaptations and reduced body temperature was proposed, at least in seasonal hibernators, for a few reasons: 1) thirteen-lined ground squirrels and the edible dormouse, both deep hibernators, appear to suppress metabolism below basal metabolic rate without a fall in T _b_ when housed in thermoneutrality ( Heldmaier & Elvert, 2004; MacCannell & Staples, 2021; Staples et al., 2022), 2) Q _10_, the ratio of the rate of activity (of an enzyme or biological system) at two different temperatures that are 10 °C apart, is typically between 2–3. However, the measured Q _10_ of the MR for some hibernators is well above 3 (see (Lawrence Chia-Huang Wang & Hudson, 1971) for an example), suggesting an active suppression of metabolism in addition to the passive effect of cooling on metabolism. Q _10_ may not be, in general, an adequate framework for analyzing MR-T _b_ relationships in heterothermic endotherms when thermal conductance can vary (see also below). Hence, changes in MR cannot be unambiguously attributed to intrinsic temperature effects on biochemical processes, as thermoregulatory responses and altered heat loss can obscure or dominate the relationship between T _b_, ambient temperature, and MR ( Heldmaier & Ruf, 1992). Notwithstanding that caveat, cellular metabolism and biochemical reactions are not impervious to the impact of temperature on their activity. One estimate for the Q _10_ of the overall metabolic rate in heterotherms is around 2.2–2.3 ( Geiser, 1988b). Given this conservative estimate for Q _10_, the cellular metabolic rate - not modified by anything but temperature - will drop about 8% per 1 °C just due to physical properties of the enzymes themselves. When fasted at an ambient temperature of 22 °C, mice reliably reach a core temperature of 25 °C or lower. From a starting T _b_ of 36 °C, this equates to a 60% metabolic saving from Q _10_ effects alone: enough to explain the 75–80% metabolic savings of *daily* torpor in the mouse ( Hudson & Scott, 1979; Nestler, 1990b). That is, the observed reductions in T _b_ during torpor are sufficient to explain the majority of the decline in MR *following* torpor entry, without invoking an additional, global coordinated suppression of metabolism. This conclusion does not exclude the possibility of active metabolic depression, nor does it deny its existence in other contexts such as hibernation; rather, it supports the hypothesis that temperature-dependent effects alone are quantitatively adequate to account for the post-entry decrease in MR in daily torpor in the mouse.

## Daily torpor: a summation of behavioural, physiological and biochemical savings

**Figure 1.**
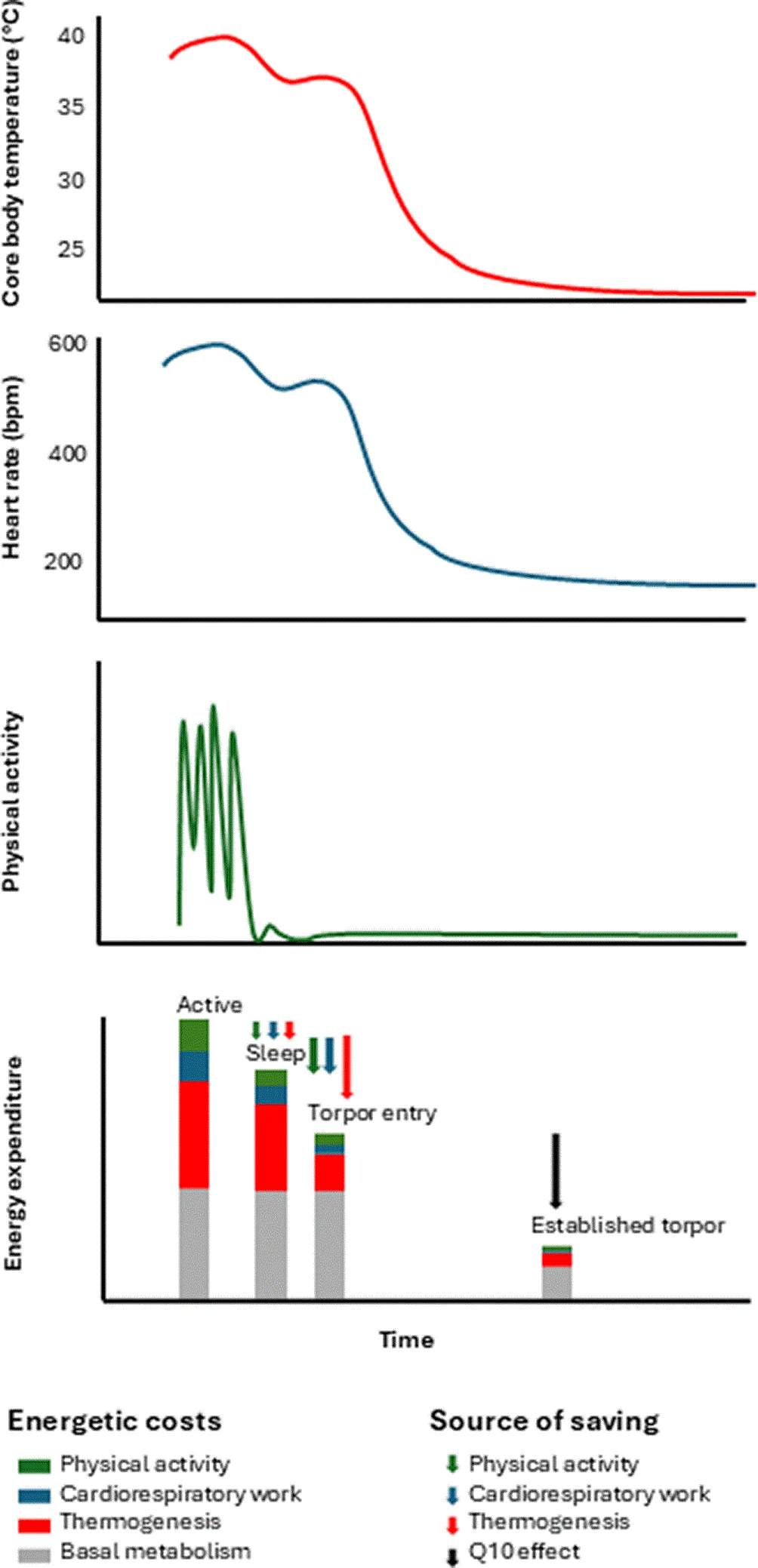
Schematic representation of torpor in relation to active and sleep states. Arrows represent source of metabolic savings associated with transition between each state. Early torpor differs from sleep primarily through reductions in thermogenesis, which provides the initial savings in torpor. Subsequently, Q10 effects dominate as temperature drops in established torpor. **Note**, this is a hypothetical proposal and is not intended to be interpreted as supportive data.

The fact that metabolic rate falls before T _b_ is one of the defining features of torpor, and our model fully captures this sequence. Crucially, this drop in MR does not reflect a systemic master mediator. Rather, we believe the “active metabolic suppression” is a consequence of continued physical inactivity and cessation of food intake associated with the sleep state, the decrease in non-shivering thermogenesis via reduced sympathetic outflow, and decreased cardiac work via elevated vagal outflow. As these energy consuming and heat-producing activities are suppressed, oxygen consumption immediately falls, followed by T _b_ as heat is lost to the environment. Without active defense of a euthermic T _b_, there is a subsequent and substantial additional reduction in metabolic rate as enzymatic activity slows in a temperature-dependent manner (see Figure 1). These changes reflect central involvement of just a few important organ systems and subsequent intrinsic slowing of cellular processes – all cells will demonstrate reduced oxygen consumption (not just muscle and BAT) via the influence of temperature on the efficiency of enzymatic catalysis of reactions in cells. The combination of the metabolic savings from these components is at least 50% of the active metabolic rate. Even in the face of heat conductance to the environment that does not change or decreases slightly with torpor ( Geiser, 2004; Sunagawa & Takahashi, 2016), T _b_ will consequently plummet. This model of daily torpor in the mouse states that the “active metabolic suppression” seen at the onset of torpor may only be a centrally-mediated inhibition of just a few select organs. Unlike hibernators, daily torpor in the mouse does not require a special mechanism or a global metabolic inhibitor. Rather, torpor in the mouse only requires a centrally generated decrease in adaptive thermogenesis in a relatively cool environment, and cessation of defense of a minimum T _b_ of 35 °C. Metabolic rate drops before T _b_, but the drop in T _b_ is critical for the nadir low metabolic state.

## Metabolic rate in ‘synthetic torpor’

Over the past few years, several experimental models that chemogenetically or optogenetically drive torpor have emerged ( Hrvatin et al., 2020; Takahashi et al., 2020; Zhang et al., 2020). This has been termed ‘synthetic torpor’ to highlight the fact that it is induced solely through central neuronal activation in the absence of a ‘natural’ trigger for torpor (i.e. fasting). Synthetic torpor provides a key paradigm for exploring the mechanisms underlying the metabolic savings associated with torpor. Whereas fasting in a *thermoneutral environment* largely prevents natural torpor ( Craig et al., 2021), it is still possible to induce synthetic torpor in a warm environment. Inducing synthetic torpor in an ambient temperature of 20 °C results in an approximately 75% reduction in oxygen consumption. In contrast, inducing synthetic torpor at a thermoneutral temperature of 32 °C results in minimal changes in T _b_ and oxygen consumption ( Takahashi et al., 2020). These observations support the argument that most of the energy savings associated with torpor in the mouse are due to physiological adjustments of thermogenesis and Q _10_ effects, rather than a system-wide signal that directly puts the brakes on cellular metabolism. Of course, it is possible that models of synthetic torpor recapitulate only certain elements of natural torpor, but to date we are unaware of any evidence from a model of synthetic torpor that demonstrates suppression of oxygen consumption in the absence of a reduction in T _b_.

We should however “bear” in mind that the mechanisms of torpor may vary across species with very different dimensions and different torpor patterns (i.e. daily versus seasonal). Unlike mice, bears are massive, with a correspondingly much-smaller SA/vol ratio. It is perhaps unsurprising then, that hibernating black bears reach a nadir T _b_ of around 30 °C and average around 33 °C. This poses a challenge if hibernating bears were to rely heavily on Q _10_ effects to reduce metabolic demand. Assuming a Q _10_ of 2.2, this would produce only a 27% reduction in oxygen consumption as the bear’s body temperature drops from 37 to 33 °C. Despite these modest reductions in T _b_, black bears achieve an approximately 75% reduction in oxygen consumption during hibernation ( Tøien et al., 2011). Furthermore, during emergence from hibernation the resting oxygen consumption remains approximately 50% of summer levels even as T _b_ reaches normal levels. Together, these observations do argue in favour of an active suppression of metabolism in larger seasonal hibernators, in contrast to the model of daily torpor in the mouse.

So where does this leave the study of torpor as a model for medical translation? Unless future work on bears can identify a ‘master metabolic regulator’, due to the larger size and relatively low SA/vol of human (akin to the bear), to achieve significant metabolic suppression would likely require active cooling alongside induction of synthetic torpor. However, there would still be merit in this approach: Q _10_ effects would generate significant metabolic savings. In this case, the aim of inducing synthetic torpor would be to modulate the central nervous system control circuits such that the normal counter-regulatory defence responses to physical cooling are not engaged. Indeed, it is likely that these counter-regulatory responses limit the usefulness of therapeutic hypothermia in previous clinical trials ( Dankiewicz et al., 2021; Dundee et al., 1953; Le May et al., 2021; Sandestig et al., 2014).

## Data Availability

No data are associated with this article.
