# Supplementary material for: Hot take on a cool topic: is there really a need for a systemic master mediator of metabolic suppression in mouse daily torpor?
Source: Wellcome Open Res. 2026 Aug 28;11:561. [Version 1] doi: 10.12688/wellcomeopenres.27516.1 (PMC13615353; doi:10.12688/wellcomeopenres.27516.1)
Supplement: Peer review history

# Reviewer report: Reviewer response for version 1

**DOI:** 10.21956/wellcomeopenres.30285.r166532

**Published:** 2026-09-30

**Authors:** Genshiro A. Sunagawa

**Affiliations:** 1 RIKEN Center for Biosystems Dynamics Research, Hyogo, Japan

Overall Impression:

 The authors present a conceptually clear and thought-provoking perspective on the energetics of daily torpor in mice. I found the quantitative framework useful. The combined effects of reduced locomotor activity, suppression of brown adipose tissue (BAT) thermogenesis, decreased cardiac workload, and temperature-dependent Q10 effects could together account for the 75–80% reduction in VO2 observed during fasting-induced torpor (FIT) in mice. As an organ-level energy budget, I think this is a reasonable and useful way to look at the problem.

 My main concern is the broader conclusion drawn from this calculation. The fact that the numbers can largely account for the reduction in whole-body VO2 does not by itself show that a “master mediator” or upstream coordinating mechanism is unnecessary. These are two different questions: one is where the reduction in energy expenditure comes from, and the other is what signal initiates and coordinates these changes across the body. The current manuscript should more clearly distinguish these levels of explanation.

 I have several specific comments.

**1. Target-Tissue Specificity Does Not Distinguish Humoral from Neural Regulation**

 The authors argue that the reduction in VO2 can largely be explained by suppression of a limited number of energetically important tissues, including BAT, skeletal muscle, and the heart, together with passive Q10 effects. Based on this, they suggest that there is little need to consider a systemic master mediator. My concern is that this conclusion goes beyond what the calculation supports.

 I am not fully convinced by this argument. It seems to assume that a circulating humoral mediator would act broadly and indiscriminately across peripheral tissues. However, that need not be the case. In classical endocrinology, circulating signals can produce highly tissue-specific effects because receptor expression, receptor density, downstream signaling pathways, and tissue accessibility differ between organs.

 Therefore, selective suppression of BAT, skeletal muscle, and cardiac metabolism would still be compatible with a circulating mediator, if its relevant receptors are selectively expressed in these tissues or in specific upstream central circuits controlling them.

 In other words, identifying which tissues account for the energy savings tells us about the downstream effectors, but not what the upstream signal is or how it acts. That signal could be neural, humoral, or part of an integrated neuroendocrine pathway. I therefore do not think that tissue-selective metabolic suppression alone argues against a systemic coordinating signal.

**2. Recalibration of Thermoregulatory Control Rather Than Simple “Cessation of Defense”**

 The authors state that torpor requires a “cessation of defense of a minimum Tb of 35 °C.” I think this wording may understate how thermoregulation continues to operate during torpor.

 Torpor is not simply an uncontrolled fall in body temperature. In hibernators, at sufficiently low ambient temperatures, animals can re-engage metabolic thermogenesis, showing that thermoregulatory control is still present but operating differently. This is also true in mouse FIT. Mathematical modeling of thermoregulation during mouse FIT also suggests that the major change is a marked reduction in thermogenic feedback sensitivity—that is, how strongly the animal responds when body temperature deviates from the theoretical set-point temperature—rather than a complete loss of thermoregulation (Sunagawa and Takahashi, 2016).

 I therefore think it would be more accurate to describe mouse torpor entry as a regulated recalibration of thermoregulatory control, rather than simply as a cessation of temperature defense at 35 °C.

**3. Temperature-Independent Physiology Beyond the Dominant Contributors to Whole-Body VO2**

 The manuscript understandably focuses on tissues that contribute substantially to resting whole-body metabolism, such as skeletal muscle, the heart, and BAT. This is reasonable when the goal is to explain whole-body VO2. However, it may miss important physiological changes in organs that make a smaller contribution to total oxygen consumption.

 For example, in Q neurons–induced hypometabolism (QIH), protection against ischemia-reperfusion kidney injury persists even when normothermia is experimentally maintained (Kyo et al., 2022). This suggests that at least some biologically important effects of a torpor-like state can occur independently of hypothermia and therefore cannot be explained only by passive Q10 effects.

 The mechanism behind this protection is still unclear. It could involve cellular metabolic suppression, altered perfusion, neuroendocrine signaling, immune modulation, or other mechanisms. My point here is not that QIH and natural FIT are equivalent. As the authors also note, they are not. Rather, this finding suggests that it may be premature to assume, based only on whole-body energy accounting, that organs with a smaller contribution to VO2 are simply passive thermal passengers during torpor.

 Conclusion:

 Overall, I think the quantitative analysis is useful and provides a clear way to explain much of the reduction in whole-body VO2 during mouse torpor. However, I do not think that this calculation alone shows that an upstream coordinating mechanism is unnecessary.

 An organ-level energy budget and an upstream neural, humoral, or neuroendocrine regulator address different questions and are not mutually exclusive.

 I therefore suggest that the authors distinguish more clearly between two ideas: a factor that directly suppresses metabolism across many or all tissues, and a coordinating mechanism that acts selectively on particular tissues or physiological processes. These are not the same hypothesis. In my view, the current analysis argues more directly against the need for a universal metabolic suppressor than against the existence of an upstream coordinating signal. Moderating the broader claim that a “master mediator” is unnecessary would strengthen the central quantitative argument.

Is the review written in accessible language?

Yes

Are all factual statements correct and adequately supported by citations?

Yes

Are the conclusions drawn appropriate in the context of the current research literature?

Partly

Is the topic of the review discussed comprehensively in the context of the current literature?

Yes

Reviewer Expertise:

Hibernation biology

I confirm that I have read this submission and believe that I have an appropriate level of expertise to confirm that it is of an acceptable scientific standard, however I have significant reservations, as outlined above.

# Reviewer report: Reviewer response for version 1

**DOI:** 10.21956/wellcomeopenres.30285.r166534

**Published:** 2026-09-25

**Authors:** Matthew D. Regan

**Affiliations:** 1 Universite de Montreal, Montreal, Québec, Canada

This was an enjoyable and thought-provoking article. It raises an interesting idea with relevance both to our understanding of torpor and its evolution among the diverse animal groups that use it, and to efforts towards inducing torpor-like states (synthetic torpor) in animals that have not evolved to use it (or, have secondarily lost their ability to use it).

 Here are some ideas that came to me while reading the article. They are not really suggestions for how the paper might be improved (it’s already published, after all); rather, they are comments to provoke thought and discussion among the authors and any torpor enthusiasts reading the article.

- I was hoping for a little more rigour with the calculations. They are essentially summed estimates of energetic savings, each with sufficiently large margins of error that the summed total can’t help but land somewhere near the 75-80% “target”. However, while the quantitative precision may be a bit limited, the general idea that a cellular-level metabolic switch is unnecessary in daily torpor is still sound. I’m actually surprised no one has bothered to calculate this before. Maybe they have?
- The authors use the term “master off switch” to describe the process by which individual cells induce an “active” metabolic depression in, say, hibernating mammals. However, I think that what they’re putting forward here – that a single process (i.e., the abandonment of 37C Tb) explains the “lion’s share” of torpor's energetic savings – actually *is* a master switch, the equivalent of heading into the basement to cut power to the whole house at the breaker instead of turning lights off individually in each room. Anyway, the use of “master off switch” for this or that scenario is semantics and not all that important, but it does underscore an important idea – how a single, central process can instigate metabolic depression in cells across the entire body (in endotherms).
- I fully agree with the authors’ interpretation of the VO2-before-Tb sequencing at torpor onset. This is often presented as proof of torpor starting with an active, temperature-independent metabolic depression at the level of the cells, but it’s also exactly what a “temperature-first” scenario would look like in an endothermic heterotherm. I feel that this is important, especially in the context of efforts towards translating this type of metabolic plasticity to humans.
- When the authors sum the energetic savings associated with fasting, sleep, zero activity, and Tb reductions from 36 to 26C, they arrive at a total savings of 75-80%, closely matching what is observed in torpid mice. These same factors are also present in the deep hibernators mentioned by the authors. The additional energetic savings observd in such species (~95% vs. 75-80%) are presented as potential evidence for an additional temperature-independent, cell-level metabolic depression mechanism. This may be true, and the authors present some ideas from the literature to support this. However, what is also true is that small hibernators like ground squirrels experience much deeper Tb reductions – to ~5C in 13-lined ground squirrels and as low as -2.9C in arctic ground squirrels (though, at these sub-zero Tb’s, the arctic ground squirrels are usually fighting steep thermal gradients in their ~ -20C burrows to maintain even -2.9C). If the authors were to have kept going and extrapolated their Q10’s to, say, 5C, would they have arrived near the 13-lined ground squirrel’s 95%? It seems close to me! In which case, is it possible that a cell-level metabolic regulator doesn’t exist in hibernators either? There’s evidence for one, of course, particularly with bears as the authors point out. But in the ground squirrels, could these pieces of evidence (e.g., PTMs of mito complexes) be consequences, not causes, of cellular metabolic depression? And/or, could they be attributable to some of the higher-level factors mentioned here that contribute to early energetic savings in mice, such as feeding cessation, movement cessation, sleep, etc? I don’t know. It seems that high-resolution sampling could be helpful here.
- More broadly, if this strategy is as physiologically straightforward as it seems, then I wonder why more endothermic animals don’t use torpor (or at least small-bodied endotherms with high SA:Vs). Maybe they do? And on the other side of the coin, I wonder why hibernators bother with the extra complexity of a cell-level metabolic depressor. Maybe they don’t? These seem like potentially useful evolutionary and mechanistic questions to ask.
- The article’s framework is quite relevant to synthetic torpor, as the current approaches for this often rely on altering thermogenesis via central activation. If a substantial fraction of the resulting metabolic depression arises due to temperature effects, then an interesting question is whether variation in Q10 among enzymes and physiological processes could ultimately limit torpor sustainability. For eg, as temp falls, perhaps different biochemical pathways will slow at different rates, progressively leading towards some mismatch or other that ultimately constrains torpor depth and/or duration. This idea could be relevant to natural and synthetic torpor.

Is the review written in accessible language?

Yes

Are all factual statements correct and adequately supported by citations?

Yes

Are the conclusions drawn appropriate in the context of the current research literature?

Yes

Is the topic of the review discussed comprehensively in the context of the current literature?

Yes

Reviewer Expertise:

Hibernation metabolism and physiology

I confirm that I have read this submission and believe that I have an appropriate level of expertise to confirm that it is of an acceptable scientific standard.

# Reviewer report: Reviewer response for version 1

**DOI:** 10.21956/wellcomeopenres.30285.r166530

**Published:** 2026-09-15

**Authors:** Matteo Cerri

**Affiliations:** 1 University of Bologna, Bologna, Italy

I found this review engaging and thought-provoking. Its principal strength is that it stimulates discussion of the physiological sequence underlying torpor entry and maintenance. The authors propose that centrally coordinated reductions in energetically demanding functions, followed by the temperature-dependent slowing of cellular processes, may account for the metabolic depression observed during fasting-induced daily torpor in mice. This is a valuable hypothesis because it challenges the perceived necessity of a dedicated systemic metabolic suppressor and encourages a more explicit examination of the mechanisms responsible for each phase of the response.

 A particularly useful distinction is that a reduction in metabolic rate preceding the fall in body temperature does not, by itself, establish the involvement of a generalized inhibition of cellular metabolism. Selective suppression of thermogenesis and other energy-consuming functions could also produce this sequence. The proposed framework therefore provides a constructive basis for debate between different interpretations of “active metabolic suppression.” I would nevertheless encourage the authors to distinguish consistently between demonstrating that a systemic suppressor is not required to explain the available observations and demonstrating that such a mechanism does not contribute. Framing the proposal as a testable hypothesis, with explicit predictions that distinguish these possibilities, would further strengthen the review.

 The scope of the argument should also remain clearly defined throughout the manuscript: it primarily concerns fasting-induced daily torpor in mice under relatively cool ambient conditions. Its applicability to other forms of torpor, seasonal hibernation, or synthetic torpor requires separate evaluation, particularly where body size, thermal inertia, and the relationship between metabolic rate and body temperature differ substantially. Within this scope, I regard the review as a valuable contribution. Its merit lies in prompting the field to clarify which mechanisms are necessary, which are sufficient, and which remain unresolved in the fundamental dynamics of torpor.

Is the review written in accessible language?

Yes

Are all factual statements correct and adequately supported by citations?

Yes

Are the conclusions drawn appropriate in the context of the current research literature?

Yes

Is the topic of the review discussed comprehensively in the context of the current literature?

Yes

Reviewer Expertise:

Physiology of torpor and synthetic torpor.

I confirm that I have read this submission and believe that I have an appropriate level of expertise to confirm that it is of an acceptable scientific standard.
